# Deep Membrane Proteome Profiling Reveals Overexpression of Prostate-Specific Membrane Antigen (PSMA) in High-Risk Human Paraganglioma and Pheochromocytoma, Suggesting New Theranostic Opportunity

**DOI:** 10.3390/molecules26216567

**Published:** 2021-10-29

**Authors:** Ondrej Vit, Mayank Patel, Zdenek Musil, Igor Hartmann, Zdenek Frysak, Markku Miettinen, Karel Pacak, Jiri Petrak

**Affiliations:** 1 BIOCEV, First Faculty of Medicine, Charles University, 25250 Vestec, Czech Republic; ondrej.vit@lf1.cuni.cz; 2 Section on Medical Neuroendocrinology, Eunice Kennedy Shriver National Institute of Child Health and Human Development, NIH, Bethesda, MD 20892, USA; mayank.patel@nih.gov (M.P.); karel@mail.nih.gov (K.P.); 3 Institute of Biology and Medical Genetics, First Faculty of Medicine, Charles University and General University Hospital, 12800 Prague, Czech Republic; zdenek.musil@lf1.cuni.cz; 4 Department of Urology, University Hospital Olomouc and Faculty of Medicine and Dentistry, Palacky University, 77900 Olomouc, Czech Republic; hartmann@email.cz; 5 Department of Internal Medicine III, University Hospital Olomouc and Faculty of Medicine and Dentistry, Palacky University, 77900 Olomouc, Czech Republic; zdenek.frysak@gmail.com; 6 Laboratory of Pathology, National Cancer Institute, NIH, Bethesda, MD 20892, USA; markku.miettinen@nih.gov

**Keywords:** neuroendocrine cancer, pheochromocytoma, paraganglioma, membrane proteomics, integral membrane proteins, Pitchfork, mass spectrometry, PSMA, theranostics

## Abstract

Pheochromocytomas and paragangliomas (PPGLs) are rare neuroendocrine tumors arising from chromaffin cells of adrenal medulla or sympathetic or parasympathetic paraganglia, respectively. To identify new therapeutic targets, we performed a detailed membrane-focused proteomic analysis of five human paraganglioma (PGL) samples. Using the Pitchfork strategy, which combines specific enrichments of glycopeptides, hydrophobic transmembrane segments, and non-glycosylated extra-membrane peptides, we identified over 1800 integral membrane proteins (IMPs). We found 45 “tumor enriched” proteins, i.e., proteins identified in all five PGLs but not found in control chromaffin tissue. Among them, 18 IMPs were predicted to be localized on the cell surface, a preferred drug targeting site, including prostate-specific membrane antigen (PSMA), a well-established target for nuclear imaging and therapy of advanced prostate cancer. Using specific antibodies, we verified PSMA expression in 22 well-characterized human PPGL samples. Compared to control chromaffin tissue, PSMA was markedly overexpressed in high-risk PPGLs belonging to the established Cluster 1, which is characterized by worse clinical outcomes, pseudohypoxia, multiplicity, recurrence, and metastasis, specifically including *SDHB, VHL,* and *EPAS1* mutations. Using immunohistochemistry, we localized PSMA expression to tumor vasculature. Our study provides the first direct evidence of PSMA overexpression in PPGLs which could translate to therapeutic and diagnostic applications of anti-PSMA radio-conjugates in high-risk PPGLs.

## 1. Introduction

Pheochromocytomas and paragangliomas (PPGLs) are rare neuroendocrine tumors arising from neural crest-derived chromaffin cells. The distinction between the two tumor types is based primarily on anatomical location. Tumors originating from the adrenal medulla are known as pheochromocytomas (PHEOs), while those arising from sympathetic or parasympathetic ganglia are known as paragangliomas (PGLs). The pathogenesis of PPGLs can be attributed to more than 20 well-identified susceptibility genes [1,2,3]. Based on mutations in fifteen of these genes and involved pathways, PPGLs are categorized into three clusters according to their molecular mechanisms: pseudohypoxia (Cluster 1), with mutations in *SDHB*, *FH*, *VHL*, *EPAS1*, *EGLN,* and several other genes of the TCA cycle or mitochondrial function; kinase signaling (Cluster 2), represented by mutated *RET*, *NF1*, *TMEM127*, *HRAS*, *FGFR1*, and *MAX*; and Wnt-altered (Cluster 3), represented by *MALMP3* and *CSDE* [2,3,4]. Clusters 1 and 2 make up a majority of the susceptibility genes causing the most common hereditary PPGLs. Mutations in these genes are important in the clinical diagnosis of these tumors, with the ability to predict and anticipate their phenotypic patterns including symptomatology, biochemistry, metastatic potential, predilection to other cancers, and therapeutic options. 

Therapeutic options for PPGL patients are currently very limited in surgically non-amenable disease and the discovery of new treatments is important in clinical care. Identifying new protein targets in these tumors suitable for both therapy and diagnosis are thus an imperative. Integral membrane proteins (especially those expressed at the cell surface) represent ideal therapeutic or imaging targets due to their functions and accessibility, as exemplified by CD20, CD30, HER 2, PD-L1, somatostatin receptors, or PSMA, to name just a few. Mass spectrometry-based proteomics has the potential to identify such tumor-overexpressed membrane proteins. However, due to their hydrophobic properties and generally low expression, specific strategies are required. Enrichment methods, selectively targeting only the hydrophilic or only the lipophilic segments of membrane proteins, are among the most effective approaches [5]. We recently developed a multi-pronged Pitchfork strategy [6] which combines glycocapture of hydrophilic glycopeptides with lectins (N-glyco-FASP, [7]) and hydrazide chemistry (SPEG, [8]) with the hpTC method selectively targeting membrane-embedded hydrophobic alpha helices [9] and a standard detergent-based proteomic analysis accessing non-glycosylated hydrophilic peptides. These four methods are complementary as they target different features of IMPs and different subsets of proteins, enabling deep profiling of membrane proteomes [6]. Here, we applied the Pitchfork strategy to the analysis of human PPGL in order to identify new potential PPGL drug targets or proteins which could serve as targets for tumor imaging tracers of PPGL.

## 2. Results

### 2.1. The Pitchfork Proteomic Analysis

We performed a detailed membrane-oriented proteomic analysis of PPGL samples using a four-pronged Pitchfork strategy [6]. The “discovery” analysis set included five PGL tumor samples, all belonging to the Cluster 1 representing PPGLs with highest risk of metastasis, multiplicity, or recurrence (patients 1, 2, and 3 harboring germline *SDHB* mutations and patients 13 and 14, both with a germline *VHL* mutation (Table 1, detailed information is provided in Table S1)). As a control chromaffin tissue, we used a pooled control adrenal medulla isolated from 10 adrenal glands.

**Figure 1:**
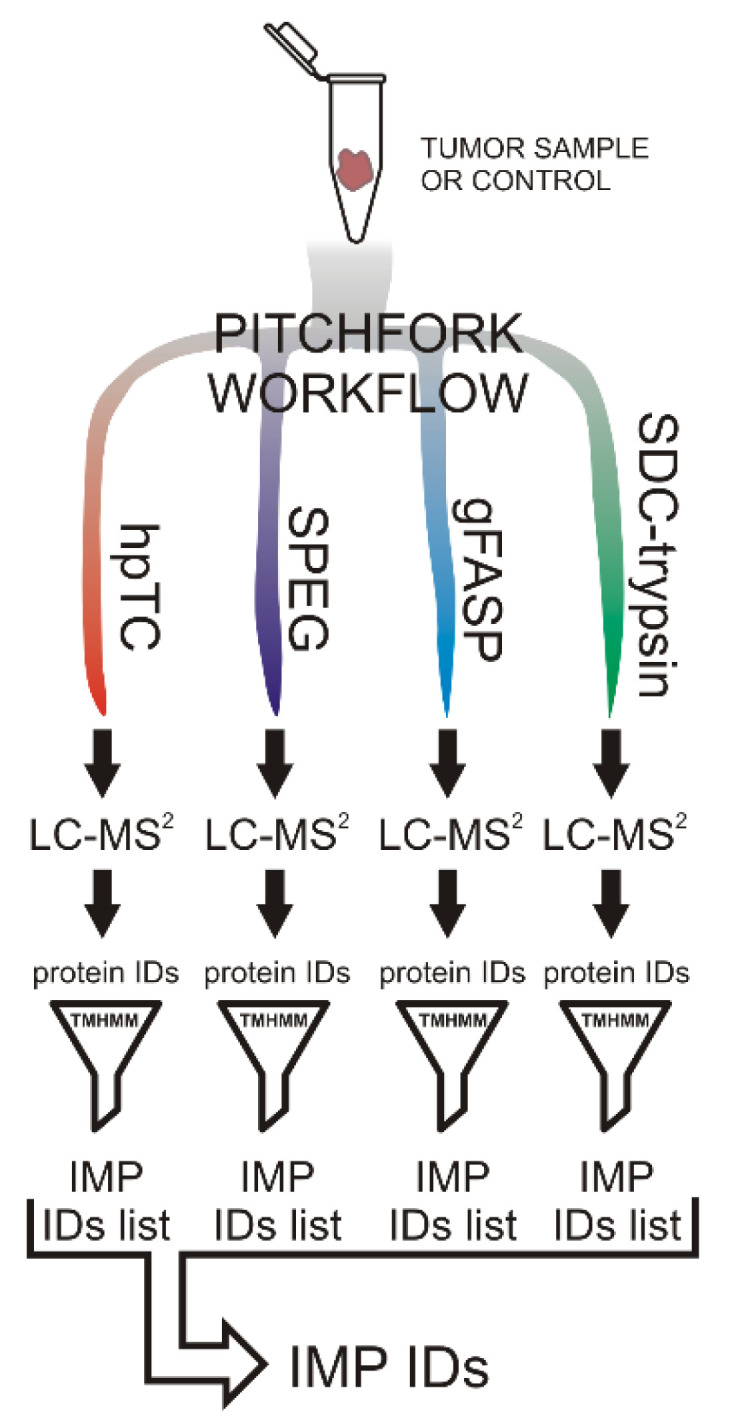
The PPGL membrane proteome analysis. Each sample was analyzed by the four methods comprising the Pitchfork strategy. Transmembrane segments of IMPs were isolated and analyzed using the hPTC method; glycosylated peptides were enriched by lectin affinity (N-glyco-FASP) and hydrazide chemistry (SPEG). Standard detergent (SDC) trypsin analysis captured non-glycosylated extra-membrane peptides. The four peptide samples were each analyzed by separate LC-MS/MS. TMHMM is a membrane protein topology prediction method based on a hidden Markov model.

Each tumor or control sample was divided and analyzed using the Pitchfork strategy comprising four parallel methods, i.e., two glycocapture (SPEG and N-glyco-FASP), the hpTC method for the enrichment of hydrophobic transmembrane segments, and a standard tissue lysate proteomic analysis [6]. Proteins identified by each method were filtered for IMPs, defined here as proteins with at least one transmembrane domain predicted by TMHMM algorithm [10]. The list of identified IMPs was then complied for each patient (Figure 1).

As we have shown previously [6], the four Pitchfork methods were complementary, providing only partially overlapping subsets of integral membrane proteins and there was no major bias with respect to the number of transmembrane segments present in the identified IMPs. The overlaps in numbers of identified proteins among the four Pitchfork methods and the distribution of identified IMPs according to the numbers of their transmembrane segments is provided in Figure S1.

**Figure 2:**
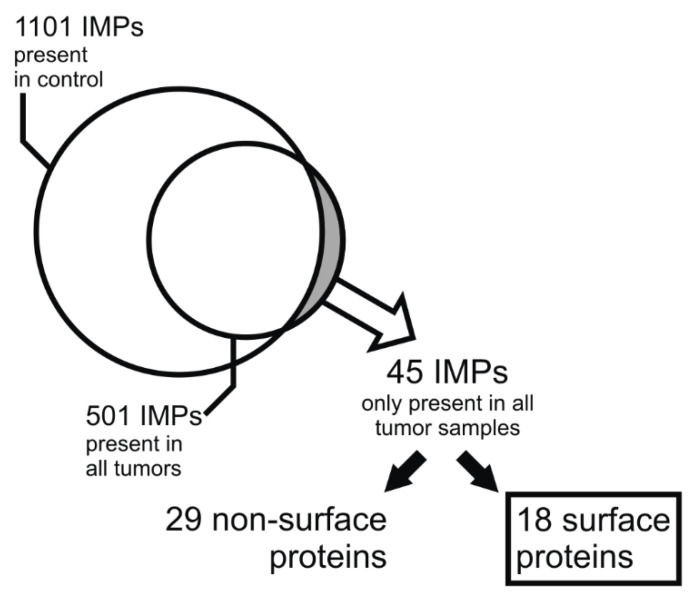
Summary of the bioinformatics workflow leading towards the selection of the candidate proteins. Forty-five IMPs were identified in all five tumors but not found in the control tissue. Of the 45 “tumor enriched” proteins, priority was given to the molecules known or expected to be present on the cell surface. Cell surface localization was determined for 18 “tumor enriched” IMPs using Surface Prediction Consensus scores.

On average, we identified 1080 IMPs in each tumor, and pooled adrenal medulla provided 1100 identified IMPs. In all five PGLs combined, we found 1856 unique IMPs. The full list of the identified IMPs is provided in Table S2. We further processed the data according to the scheme depicted in Figure 2. Of the 1856 tumor IMPs, we compiled a list of 501 proteins common for all five PGL samples and compared it to the list of proteins identified in the control adrenal medulla. This provided a group of 45 “tumor enriched” IMPs, i.e., membrane proteins present in all five PPGLs but not detected in the control pooled adrenal medulla sample (Table S3). Presence in all five PGLs and concurrent absence in the pooled control adrenal medulla sample indirectly suggested higher expression of these IMPs in the PPGLs. Looking for potential drug targets or proteins exploitable for tumor imaging, we considered their subcellular localization in addition to their relative overexpression. Since the presence of a protein in the plasma membrane theoretically enables the best access of a drug or an imaging tracer, we narrowed down the list of “tumor enriched” proteins to only those present on the cell surface. The filtration was performed using Surface Prediction Consensus (SPC) scores, which predict the likelihood of the protein being localized on cell surface [11] (Figure 2). Of the 45 “tumor enriched” proteins, 18 reached SPC score 3 or 4, suggesting a high probability of their localization on the cell surface/in the plasma membrane (Table 2).

Among the 18 “tumor enriched” cell surface IMPs listed in Table 2, one protein clearly stood out with respect to its diagnostic or therapeutic potential. Glutamate carboxypeptidase 2, better known as prostate-specific membrane antigen (PSMA), is the approved and clinically used theranostic target in advanced prostate cancer. Radioconjugates of ligands which bind to PSMA protein overexpressed on the surface of prostate have been routinely used for tumor imaging (^68^Ga-PSMA-11 ligand for PET/CT scanning), and, more importantly, the therapeutic ^177^Lu-PSMA-617 conjugate has been recently shown to reduce death rate and prolong lives of the patients with castration-resistant prostate cancer (CRPC) in phase III clinical study (VISION) [12,13].

Identification of PSMA in all five PGLs along with its absence in the control adrenal medulla sample suggested PSMA overexpression in PPGL. If confirmed, this could open the door for the application of the theranostic anti-PSMA radioconjugates in some PPGL patients. Therefore, we focused our efforts on an independent confirmation of PSMA overexpression.

**Figure 3:**
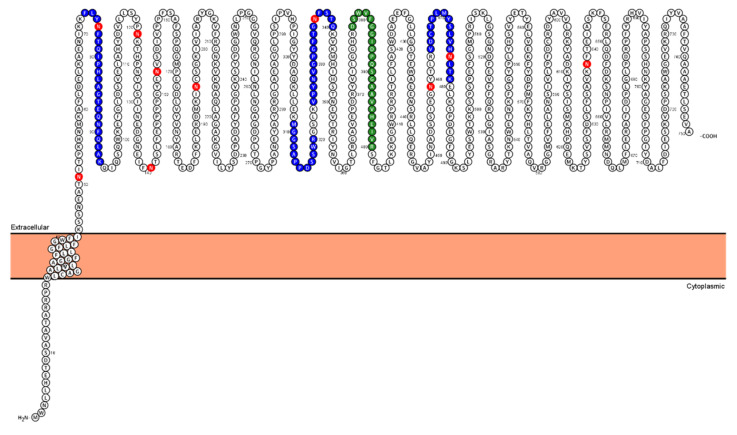
Topology of PSMA with mapped identified peptides. The 5 identified peptides were mapped to the PSMA sequence. The 4 peptides identified by the glycocapture methods N-glyco-FASP and SPEG are marked in blue, and the single peptide found by the standard SDC-trypsin approach is shown in green. The red Asn residues are N-glycosylation sites annotated in UniProt. The visualization was created by Protter software [14].

We first manually verified the MS-based identifications of PSMA in the proteomic study. PSMA was identified by five unique peptides, four being provided by glycocapture and one by standard tryptic digest (Figure 3). Annotated MS/MS spectra are shown in Figure S2.

### 2.2. Verification of PSMA Overexpression

**Figure 4:**
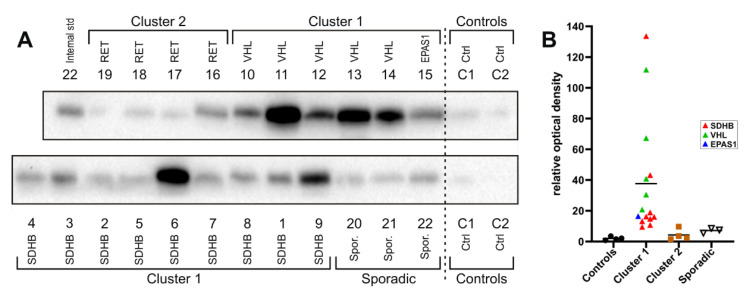
Western blot and densitometry of PSMA signal. (**A**) Western blots of all 22 tumor samples and 2 controls (pooled adrenal medulla). Lysate samples, each representing 30 micrograms of protein, were loaded. To normalize the PSMA signal intensity between the 2 membranes, sample 22 was present on both membranes and used as an internal standard. Loading control: Due to heterogeneity of tumors and individuals, standard loading control based on “housekeeping” proteins (GAPDH, Tubulin, Actin) could not be used, as their antibody-detected levels varied significantly among the individual samples. To solve this problem, we ran each gel in duplicate; one gel was used for Western blotting and the second was stained by Coomassie Brilliant Blue (CBB), and the total optical density was determined for each lane to ensure equal protein loading. (**B**) Normalized optical densities of the PSMA signal from the Western blots are plotted for individual samples being divided according to the respective PPGL clusters (Controls, Cluster 1, Cluster 2, Sporadic). Colors indicate the underlying mutated genes in Cluster 1 PPGLs.

Next, we determined the relative expression of PSMA by Western blotting in a set of 22 PPGL samples (Table 1) using a specific antibody (clone GCP-04, [15]). The set included 11 PGLs and 11 PHEOs representing Cluster 1 (pseudohypoxia, n = 15), Cluster 2 (kinase signaling, n = 4), and apparently sporadic tumors (n = 3). Cluster 1 was represented by PPGLs of patients with germline mutations in *SDHB* (n = 9), germline or somatic *VHL* mutations (n = 5), and a somatic *EPAS1* mutation (n = 1). All tumors belonging to Cluster 2 (n = 4) carried a germline *RET* mutation. Apparently sporadic PPGLs (n = 3) were defined as tumors from patients without a germline mutation in the known susceptibility PPGL genes together with no known previous or family history of PPGL (for detailed patient information, see Table S1). Five of the twenty-two samples were identical to those used in the initial “discovery” proteomic analysis. (i.e., patients 1–3, 13, and 14). As a negative control, we used two independent samples of pooled healthy chromaffin tissue, each generated by combining the isolated adrenal medulla dissected from 10 different individuals (Figure 4).

The immunoblotting showed massively increased expression of PSMA in PPGLs from Cluster 1 harboring *SDHB, VHL*, or *EPAS1* mutations compared to the control adrenal medulla sample (on average, 18-fold increase compared to control, range 4–55-fold). Expression of PSMA in Cluster 2 PPGLs (all with *RET* mutations) and apparently sporadic tumors was only mildly increased (1.5–4-fold) compared to the control adrenal medulla sample (Figure 4).

### 2.3. Immunohistochemistry

In addition to prostate cancer, several reports showed PSMA upregulation in other tumors, predominantly localized to tumor neovasculature [16,17,18,19]. To determine the PSMA localization in PPGL, we performed immunohistochemical staining on the same set of tumor samples, with the exception of three samples (Nos. 4, 9, and 15) which were not available. Of the 19 PPGL samples, 10 tumors stained positive for PSMA (8 out of 12 Cluster 1 PPGLs and two sporadic tumors). Nine samples were characterized as PSMA-negative, including all four samples belonging to the Cluster 2 PPGLs, one sporadic tumor, and four Cluster 1 PPGLs.

**Figure 5:**
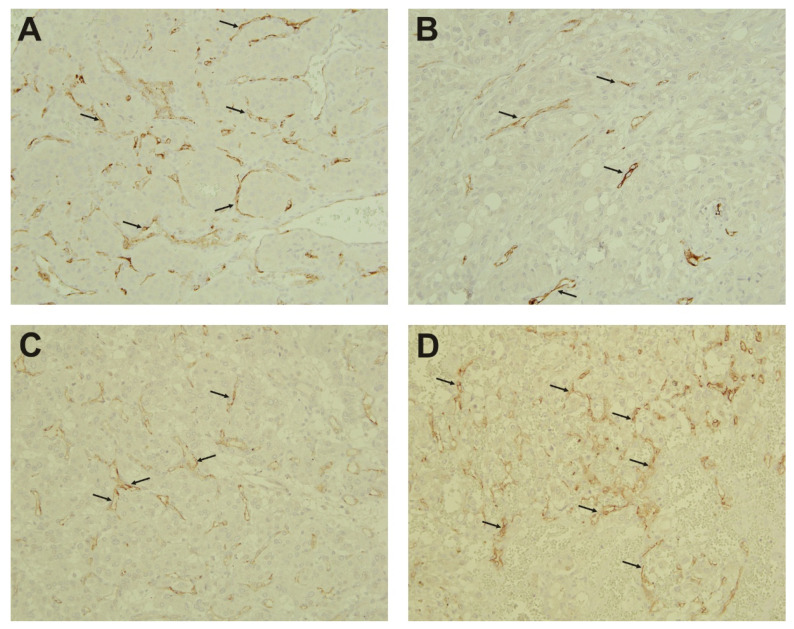
Immunohistochemistry. PSMA positivity and localization in Cluster 1 PPGLs. (**A**) *SDHB*-mutated bladder PGL (patient 1). (**B**) *SDHB*-mutated mediastinal PGL (patient 6). (**C**) *VHL*-mutated pheochromocytoma (patient 10). (**D**) *VHL*-mutated pheochromocytoma (patient 12). Brown PSMA staining in endothelial lining of tumor vessels is indicated by arrows.

Figure 5 shows examples of PSMA-positive tumors. Samples 1 and 6 represent two *SDHB*-mutated PGLs (Figure 5A,B) and two *VHL*-mutated PHEOs (patients 10 and 12, Figure 5C,D). The stained samples showed PSMA expression (brown staining indicated by the arrows) in the endothelial lining of tumor vessels but not in the tumor cells, being in agreement with previous observations in various solid tumors other than prostate cancer (Figure 5).

## 3. Discussion

### 3.1. PSMA—An Effective Theranostic Target

PSMA is a type II transmembrane glycoprotein with folate hydrogenase and carboxypeptidase activities coded by *FOLH1* gene. PSMA has a large glycosylated extracellular domain responsible for its enzymatic activity, a transmembrane segment, and a short intracellular domain. The short cytosolic tail contains an internalization motif and interacts with various proteins enabling PSMA endocytosis [20]. The molecule is overexpressed in prostate cancer where it correlates with tumor aggressivity and vascularization and it is used as a target for radioconjugates for prostate cancer imaging (FDA approval in 2020) and recently also for therapy of advanced prostate cancer [12,13]. In addition to prostate cancer, PSMA expression has been demonstrated in several solid tumors [16,17,18,19].

Using a hypothesis-free deep proteomic profiling of PGL membrane proteome, we identified a subset of “tumor enriched” IMPs including PSMA. Antibody-based verification was performed in a panel of 22 PPGLs representing two of the three main PPGL clusters [2,4]: Clusters 1 and 2, which can be attributed to pathogenesis in nearly 90–95% of all heritable PPGLs, including primary and metastatic tumors. The antibody-based verification confirmed massive (mean 18-fold, range 4–55-fold) overexpression of PSMA in Cluster 1 PPGLs. PSMA expression in the Cluster 2 PPGLs (all with *RET* mutations) and apparently sporadic PPGLs was only mildly increased (1.5–4-fold). Additionally, using IHC, we localized PSMA protein to endothelial cells in tumor vasculature.

Thus, we provide here the first direct evidence of PSMA and its overexpression in the subset of PPGLs relative to control chromaffin adrenal tissue. However, it should be noted that several previous coincidental findings pointed toward the possible expression of PSMA in isolated PHEO [21] or PGL [22,23,24] cases.

The marked upregulation of PSMA in the “pseudohypoxia driven” Cluster 1 PPGLs with pathogenic *SDHB*, *VHL*, or *EPAS1* mutations opens a new door toward the existing theranostic application of already formulated and approved PSMA-targeted radioligands utilized in metastatic castration-resistant prostate cancer. Thus, PET/CT imaging with Gallium-68 chelated to a peptide, antibody, or a small molecule recognizing PSMA (^68^Ga-PSMA) could be used for diagnostic imaging of PPGLs overexpressing PSMA. Using the same concept, it could be used for targeted therapy by switching the radiometal to Lutetium-177 forming ^177^Lu-PSMA-617. The conjugate radioligand, ^177^Lu-PSMA-617 has been recently shown to reduce death rate and prolong the lives of the patients with metastatic castration-resistant prostate cancer in a phase III clinical study (VISION) [12]. Our findings establish the basis for further studies to look at pinpointed radiotherapeutic approaches by alternating beta-emitting molecules ^177^Luteitium to alpha-emitting particles such as ^223^Radium and ^225^Actinium. Alpha emitters conjugated to an anti-PSMA molecules have the potential to deliver higher energy at a shorter wavelength, benefitting smaller metastatic PPGL lesions [25].

*SDHB*-related PPGLs have been shown to have a poor prognosis and a high metastatic potential [26,27,28]; therefore, new therapies that can stabilize the disease and provide a better outcome for patients are of high clinical value. Similarly, the identification of PSMA overexpression in other Cluster 1 PPGLs, specifically *VHL*-mutated, have similar potential. While *VHL*-driven tumors do not carry the same high metastatic risk as *SDHB*-driven, there is a 25% hereditary predisposition to PPGL which makes these PSMA findings important for the patient and family at risk of disease. *VHL* syndrome predisposes PPGL patients to the risk of concomitant life-threatening cancers such as pancreatic neuroendocrine tumors, renal cell carcinoma, and central nervous system hemangioblastoma [29]. Further studies on each of these *VHL*-related cancers should be performed to uncover whether PSMA is overexpressed in these tumors.

The success of PSMA-labeled radiotherapy in metastatic castration-resistant prostate cancer has the potential of being expanded to other tumors expressing PSMA. Our study shows that Cluster 1 PPGLs markedly overexpress PSMA compared to the healthy adrenal medulla, with the most likely location being in tumor neovasculature. This finding warrants a larger inspection of PSMA overexpression in PPGLs and other neuroendocrine tumors. A possible theranostic target such as PSMA is a beneficial finding for PPGL patients, given the clinical impact of current radiolabeled agents such as ^68^Ga-DOTATATE [30,31,32,33] and ^177^Lu-DOTATATE (ClinicalTrials.gov Identifier: NCT03206060) in Cluster 1 PPGL patients. In current practice, radionuclide imaging specific to localizing PPGLs are reserved to FDA-approved ^68^Ga-DOTATATE and ^123^I-metaiodobenzylguanidine (MIBG). However, there are PPGLs that do not display positivity on either of these imaging modalities; these patients could benefit from ^68^Ga-PSMA conjugates based on our initial findings. PSMA PET imaging would also cover the whole body, and combined with CT scans, would help localize metastatic and multiple lesions in various anatomic regions, which are two features important in the phenotypic pattern of Cluster 1.

### 3.2. PSMA Function

The intrinsic enzymatic (or other) role of PSMA in tumor growth has remained unclear and, therefore, rather unexploited. In the brain, PSMA catalyzes glutamate synthesis from neuropeptide NAAG, promoting neural transmission [34], while in the intestine, PSMA may participate in folate absorption [35,36]. In pancreatic cancer, PSMA is overexpressed on the tumor cells, where its positive role in folate uptake and cell proliferation has been documented [37]. PSMA expression was also found in several other solid cancers (glioblastoma, kidney, liver, thyroid, lung, and breast cancers), but always localized predominantly in tumor neovasculature, not in normal vessels [16,17,18,19]. In agreement, we have shown PSMA in PPGLs to be localized in the tumor vasculature. The intrinsic role of PSMA in tumor vascular endothelia is far from being fully established, but PSMA’s role in endothelial cell sprouting has been proposed and PSMA-mediated cleavage of laminin has been shown to produce pro-angiogenic peptides [38,39,40]. These observations suggest that the inhibition of PSMA activity could lead to the development of novel anti-angiogenic strategies. Exclusive expression of PSMA in tumor neovasculature of solid tumors, but not in normal vessels, highlights PSMA not only as a target for radio-ligand or other conjugate therapy but also as an enzyme whose specific inhibition may have anti-angiogenic effects.

### 3.3. Other Identified Putative Targets Overexpressed in PPGLs 

Our study using the Pitchfork strategy demonstrated that a comprehensive hypothesis-free membrane proteomic strategy can identify tumor-overexpressed membrane proteins—molecules with therapeutic or diagnostic potential. In addition to PSMA, 17 other cell surface “tumor enriched” IMPs were identified in the initial proteomic analysis. Their overexpression in PPGL tumors must be independently confirmed; however, their provisional overexpression in PPGL may help to direct future studies looking for new therapeutic or diagnostic targets. Among the 17 candidates, at least five proteins clearly stand up for their established association with cancer. Namely, CLEC14A, CD93, endoglin, and ANTXR1 (alias TEM8) have shown to be overexpressed in several cancers. All four molecules are predominantly endothelial proteins involved in tumor neovascularization, and as such are being considered and validated as promising drug targets or targets for tumor imaging [41,42,43,44]. Similarly, sulfhydryl oxidase 1 (QSOX1) has been intensively studied in relation to cancer. QSOX1 over-expression in breast and prostate cancers correlates with tumor invasiveness [45,46]. QSOX1 contributes to metastasis-related ECM rearrangement in breast cancer, and anti-QSOX1 antibodies and LMW inhibitors suppressed the growth of cancer cells in mouse models [47,48].

## 4. Materials and Methods

### 4.1. Adrenal Medulla Chromaffin Tissue Samples

Adrenal glands were obtained from patients undergoing curative adrenalectomies due to primary aldosteronism or radical nephrectomies including adrenal glands due to the presence of renal tumors. All adrenal samples were inspected by a skilled pathologist for any characteristics consistent with pathologic changes. Portions of adrenals were immediately stored and frozen at −80 °C. The adrenal medulla was manually and carefully dissected under stereomicroscope. The isolated chromaffin medulla tissue was stored at −80 °C. The two pooled samples of chromaffin tissue were generated by combining the isolated adrenal medulla dissected from 10 different individuals each, controlled for sex and surgical indication (nephrectomy vs. adrenalectomy). All subjects gave their informed consent for inclusion before they participated in the study. The study was conducted in accordance with the Declaration of Helsinki, and the protocols were approved by the Ethics Committee of Faculty Hospital and Medical Faculty of Palacky University in Olomouc (approval no. 13/14) and by the Ethics Committee of General University Hospital and First Faculty of Medicine, Prague (approval no. 120/14).

### 4.2. Pheochromocytoma and Paraganglioma Samples

PPGLs included in this study were surgically resected from patients enrolled and evaluated under a protocol approved by the Eunice Kennedy Shriver National Institute of Child Health and Human Development Institutional Review Board (ClinicalTrials.gov Identifier: NCT00004847). Informed consent was obtained from all patients for clinical, genetic, biochemical, and imaging studies performed as part of the investigation. The study was conducted in accordance with the Declaration of Helsinki. Surgical intervention of these tumors occurred between August 2006 and December 2017, which were all confirmed on histopathology to be PHEO or PGL. A total of 22 PPGL samples were included in the study. These samples included 8 primary PGLs, 11 primary PHEOs, 1 recurrent PHEO, 1 recurrent and metastatic PGL, and 1 metastatic PGL. Clinical findings that are known to potentiate aggressive disease, such as size, biochemical secretion, and age [26,49,50,51,52,53], are summarized in Supplementary Table S1 and in abbreviated Table 1. The size of tumors was determined from measurements of the gross specimen after surgical resection. Biochemical phenotypes were classified into the following groups: noradrenergic, elevation in plasma norepinephrine and/or normetanephrine; adrenergic, elevation in plasma epinephrine and/or metanephrines; and dopaminergic, elevation in plasma dopamine and/or methoxytyramine. One patient (No. 22) was classified based on the measurement of urinary catecholamines and metanephrines due to scheduling logistics preventing the acquisition of plasma biochemical testing prior to surgery. Fresh tumor samples were stored and frozen within 1 h of surgical resection at −80 °C. Slide recuts (5 microns) of the tumor samples for IHC staining were prepared and acquired from paraffinized tissue blocks by the NCI Laboratory of Pathology (Bethesda, MD, USA).

### 4.3. Mutation Screening

All patients underwent genetic testing for PPGL susceptibility genes as a part of their NIH clinical research evaluation or at their referring institution. Germline and somatic mutations in PPGL susceptibility genes are summarized in Table 1 and extended Supplementary Table S1. Of the 22 patients, 18 had germline mutations attributed to PPGL susceptibility genes. Nine patients were found to have *SDHB* mutations, five were found to have *VHL* mutations, and four were found to have *RET* mutations. One patient (No. 15) carried a somatic mutation in *EPAS1*/*HIF2A* gene and was negative for 19 gene germline mutation testing (National Heart, Lung, and Blood Institute (NHLBI) testing panel: *RET*, *MAX*, *VHL*, *SDHA*/*B*/*C/D/AF2*, *TMEM127*, *NF1*, *KIF1Bbeta*, *EGLN1*, *EGLN2*, *K-RAS*, *IDH1*, *IDH2*, *FH*, *MDH2*, and *HIF2A*). Three patients (Nos. 20, 21, and 22) were found to be negative for mutations in well-known PPGL susceptibility genes. Tumors from two of these patients (Nos. 20 and 22) were concluded to be apparently sporadic based on negative family history for PPGL and negative genetic testing, the first including 8 genes (Genetic PPGL Mayo Testing: *RET, MAX, VHL, SDHB/C/D/AF2,* and *TMEM127*) and the second including 19 genes (NHLBI testing: *RET, MAX, VHL, SDHA/B/C/D/AF2, TMEM127, NF1, KIF1Bbeta, EGLN1, EGLN2, K-RAS, IDH1, IDH2, FH, MDH2*, and *HIF2A*). The tumor from the third patient (No. 21) was also considered as apparently sporadic after negative genetic testing for *RET* (Genetic PPGL Mayo Testing) due to clinical suspicion of multiple endocrine neoplasia (MEN), despite having a somatic *CTNBB1* somatic mutation (currently not implicated in hereditary PPGL) somatic mutation. For the remaining three patients (Nos. 10, 13, and 16), genetic testing reports were not available, but their clinical presentation and gene mutations listed and described in clinical notes were conclusive for mutations attributed to PPGLs. Only two tumor samples (Nos. 15 and 21) were tested for somatic mutations.

### 4.4. Proteomic Analysis

Tumor samples and pooled control adrenal tissue were pulverized under liquid nitrogen and aliquoted. Five PGL samples (patient Nos. 1, 2, and 3 with germline *SDHB* mutations and samples 13 and 14 from patients with germline *VHL* mutations) and one pooled control adrenal medulla sample underwent 4 parallel analyses, using two glycocapture methods (SPEG, N-glyco-FASP), a method for the enrichment and extraction of hydrophobic transmembrane peptides (hpTC), and a standard detergent trypsin-based analysis.

#### 4.4.1. SPEG

The method was performed as by Vit et al. [6]. Briefly, crude membrane fractions were isolated from about 100 mg of tissue homogenized in liquid nitrogen and solubilized in a high-salt buffer (2 M NaCl and 1 mM EDTA in 10 mM HEPES-NaOH, pH = 7.4), sonicated and centrifuged at 20,000× *g* for 30 min at 4 °C. The pellet was re-extracted by passing through a 20 G hypodermic needle in 100 mM Na_2_CO_3_ with 1 mM EDTA, shaken for 30 min on ice, centrifuged, re-extracted, and incubated on ice one more time. The membrane fraction was solubilized and digested according to Masuda et al. [54]. The membrane pellet was resuspended in 5% sodium deoxycholate (SDC) in 100 mM NH_4_HCO_3_, incubated for 15 min at room temperature, and homogenized by pipetting and sonication and insoluble debris was pelleted at 15,000× *g* (room temperature). The proteins were reduced with 20 mM dithiothreitol and alkylated with 45 mM iodoacetamide, and the sample was diluted to a final concentration of 1% SDC and 50 mM NH_4_HCO_3_ prior to trypsin digestion. Trypsin (Promega) was added at a 1:50 trypsin: protein ratio and the sample was incubated at 37 °C overnight. The sample was acidified with trifluoroacetic acid (TFA) to pH < 3, and SDC was removed by extraction with ethyl acetate added at 1:1 volume by vigorously shaking for 1 min and subsequent centrifugation at 15,000× *g* for 2 min; this process was repeated 3–4 times. The peptide sample was desalted using OptiTrap™ columns (Optimize Technologies) according to the manufacturer’s instructions. SPEG itself was carried out according to Sun et al. [55], with minor modifications we introduced previously [6]. The desalted peptide sample was solubilized and diluted in 500 µL of Affi-Gel Hz Coupling Buffer (Bio-Rad). The sample was oxidized with 10 mM NaIO_4_ for 1 h in the dark, and the oxidation was quenched by the addition of 20 mM Na_2_S_2_O_3_ for 10 min. Affi-Gel Hz Hydrazide Gel beads (Bio-Rad) were washed with ddH_2_O and twice in the Coupling Buffer, mixed with the sample and rotated overnight at room temperature. The beads were washed with 1.5 M NaCl, 80% acetonitrile, and 50 mM NH_4_HCO_3_, and bound peptides were cleaved in 25 µL of 50 mM NH_4_HCO_3_ with 3 units of peptide N-glycosidase F (PNGase F, Roche) at 37 °C overnight. The supernatant was collected by centrifugation along with additional washes (2 × 50 µL of 50 mM NH_4_HCO_3_, 40 µL of 0.5 M NaCl, and 40 µL of 80% acetonitrile) and the sample was desalted before LC-MS/MS.

#### 4.4.2. N-glyco-FASP

The membrane fraction protein digest was prepared the same way as in SPEG. The glycocapture itself was carried out according to Zielińska et al. [7]. The desalted peptide sample was solubilized and diluted with binding buffer (0.5 M NaCl, 1 mM MnCl_2_, 1 mM CaCl_2_, 20 mM tris-HCl, pH = 7.6) and mixed with 100 μg of wheat germ agglutinin (WGA), 100 μg of concanavalin A (ConA), and 80 of μg Ricinus communis agglutinin I (RCA_120_). The mixture was incubated for 1 h on a rocker in a 10 kDa ultrafilter device (Microcon Ultracel-10 Membrane, 10 kDa), washed 4 times with 200 μL of binding buffer and twice with 50 mM NH_4_HCO_3_, and incubated in 40 μL of 50 mM NH_4_HCO_3_ with 2 units of PNGase F at 37 °C overnight. The deglycosylated peptides were collected by centrifugation and 3 additional washes (twice with 50 μL of 50 mM NH_4_HCO_3_ and once with 40 μL of 0.5 M NaCl) were mixed with the supernatant. The peptides were desalted before LC-MS/MS.

#### 4.4.3. The High pH Trypsin Cyanogen Bromide (hpTC) Method 

The method was performed in line with the published protocol [9]. Briefly, 100 mg of tissue was pulverized under liquid nitrogen. The resulting powder was then homogenized in hypotonic buffer (10 mM NaCl, 2 mM MgCl_2_, 10 mM HEPES, pH = 7.4) after 15 min incubation on ice by passing through a 20 G hypodermic needle. Cell debris was centrifuged at 500× *g* and the lysate was treated with 120 Kunitz units of bovine deoxyribonuclease I with 25 mM MgCl_2_ and 5 mM CaCl_2_ for 30 min at 37 °C. The membranes were pelleted at 20,000× *g*, 4 °C and shaken in 100 mM Na_2_CO_3_ with 1 mM EDTA for 30 min on ice, this step being repeated once more. The membrane pellets were resuspended in 50 mM ammonium bicarbonate with 20 µg trypsin (Promega) and incubated at 37 °C overnight. The sample was again washed twice with Na_2_CO_3_ and pelleted, and the sample was snap-frozen on dry ice and thawed three times between the washes. The membranes were solubilized in 70% trifluoroacetic acid (TFA) with CNBr (20 mg/mL) and incubated in the dark at room temperature overnight. The suspension was evaporated, and the peptides were solubilized in 80% acetonitrile, 10% isopropanol, and 5% formic acid (FA), and diluted 1:10 with 0.5% FA. The sample was desalted and delipidated on an OptiTrap™ column (Optimize Technologies): washed once with 0.5 mL 0.5% FA, 5 times with 0.5 mL dichloromethane with 0.5% FA, again with 0.5 mL 0.5% FA, and eluted with 80% acetonitrile, 10% isopropanol, and 0.5% FA [9,56].

#### 4.4.4. SDC-Trypsin 

About 10 mg of tissue (tumor or pooled adrenal medulla) was homogenized in liquid nitrogen, solubilized, and digested according to Masuda [54]. The material was resuspended in 5% SDC in 100 mM NH_4_HCO_3_ and digested the same way as the membrane fraction in SPEG and N-glyco-FASP (see above).

#### 4.4.5. nLC-MS/MS Analysis

The peptide samples were separated on a reversed phase nano column (EASY-Spray column(Thermo Fisher Scientific, Waltham, MA), 50 cm × 75 µm ID, PepMap C18, 2 µm particles, 100 Å pore size). Mobile phase buffer A was composed of water, 2% acetonitrile, and 0.1% formic acid. Mobile phase B was composed of 80% acetonitrile and 0.1% formic acid. Samples were loaded onto the trap column (Acclaim PepMap300(Thermo Fisher Scientific, Waltham, MA, USA), C18, 5 µm, 300 Å Wide Pore, 300 µm × 5 mm, 5 Cartridges) for 4 min at 15 μL/min, with a loading solution composed of water, 2% acetonitrile, and 0.1% trifluoroacetic acid. After 4 min, the valve was switched, and mobile phase B was increased from 4% to 35% B in 120 min at 300 nL/min, followed by a wash with 75% B 5 min at 400 nL/min, and then 4% B for 5 min until the end of the run (for all samples except hpTC). For the hpTC samples, the mobile phase B was increased from 4% to 50% B in 120 min at 300 nL/min, followed by a wash with 75% B 5 min at 400 nL/min, and then 4% B for 5 min until the end of the run. Eluting peptide cations were converted to gas-phase ions by electrospray ionization and analyzed on a Thermo Orbitrap Fusion (Thermo Fisher Scientific, Waltham, MA, USA) (Q-OT-qIT, Thermo). Survey scans of peptide precursors from 350 to 1400 m/z were performed at 120K resolution (at 200 m/z) with a 5 × 10^5^ ion count target. Tandem MS was performed by isolation at 1.5 Th with quadrupole, HCD fragmentation with a normalized collision energy of 30, and rapid scan MS analysis in the ion trap. The MS^2^ ion count target was set to 10^4^ and the max injection time was 35 ms. Only the precursors with charge state 2–6 were sampled for MS^2^. The dynamic exclusion duration was set to 45 s with a 10 ppm tolerance around the selected precursor and its isotopes. Monoisotopic precursor selection was turned on. The instrument was run in top speed mode with 2 s cycles [57].

#### 4.4.6. Proteomic Data Processing

For protein identification, MS raw data files were analyzed using MaxQuant v1.6.0.7. The data were searched against the human subset of the Swiss-Prot database (20,395 sequences). The search settings differed between the methods. Trypsin/P with 2 max missed cleavage sites was selected in all methods; in addition, CNBr (defined as cleavage C-terminal to methionine) with 2 max missed cleavage sites was selected in the hpTC analyses. N-terminal protein acetylation and oxidation of methionine were included as variable modifications for all methods. For SPEG, asparagine deamidation to aspartate was included, and for hpTC, the change of methionine (C-terminal to any peptide) to homoserine lactone (Met-48.003) and homoserine (Met-29.993) was included. For all methods except hpTC, carbamidomethylation of cysteine was used as a fixed modification. The false discovery rate (FDR) was set to 1% for both proteins and peptides.

The identified proteins’ FASTA sequences from each separate LC-MS/MS analysis (4 per sample— N-glyco-FASP, hpTC, SPEG, SDC-trypsin) were submitted to TMHMM Server v. 2.0 (http://www.cbs.dtu.dk/services/TMHMM/, [10]) to identify integral membrane proteins (IMPs) and to filter out non-membrane proteins. This resulted in 4 sub-lists of IMPs (corresponding to N-glyco-FASP, hpTC, SPEG, and SDC-trypsin) for each sample. A final list of all IMPs identified was then compiled for each sample from the four sub-lists of IMPs. A set of 501 common IMPs, identified concurrently in all 5 tumors, was established. The list of “tumor enriched” proteins is composed of IMPs expressed in all tumors but not found in the control tissue. To determine whether an IMP is localized on the cell surface, Surface Protein Consensus (SPC) scores were searched using the SurfaceGenie web application at https://gundrylab.shinyapps.io/surfacegenie/ [21]. Proteins with SPC scores equal to or larger than 3 were then considered as cell surface proteins.

### 4.5. SDS-PAGE and Western Blotting

The tissue lysates for SDS-PAGE and Western blotting were prepared the same way as samples for SDC-trypsin prior to reduction, alkylation, and digestion. The centrifuged lysates in 5% SDC were mixed with Laemmli sample buffer (final 2% SDS, 10% glycerol, 5% 2-mercaptoethanol). Electrophoreses were run in a Mini-Protean TetraCell module (Bio-Rad) in Tris/Glycine/SDS buffer (Bio-Rad). Due to heterogeneity of tumors and individuals, standard loading control based on “housekeeping” proteins (GAPDH, Tubulin, Actin) could not be used as their antibody-detected levels varied significantly among the individual samples. To solve this problem, we ran each gel in duplicate. One gel was used for Western blotting and the second was stained by Coomassie Brilliant Blue (CBB), and the total optical density was determined for each lane to ensure equal protein loading. The gels were then transferred to Trans-Blot Turbo Mini 0.2 µm PVDF membranes in the Trans-Blot Turbo Transfer System (Bio-Rad), washed in PBS with 0.1% Tween 20 (PBST), and blocked in 5% (*w*/*v*) skim milk in PBST for 30 min. The membranes were washed 3 times/5 min in PBST sealed in small volume with primary anti-PSMA antibody (GCP-04, Exbio) and incubated for 15 min at room temperature and at 4 °C overnight. The membranes were washed 3 times/10 min in PBST, incubated with horseradish peroxidase-conjugated secondary antibody (m-IgGκ BP-HRP, sc-516102, Santa Cruz) for 30 min, and washed 3 times/10 min in PBST and once in PBS. The membranes were incubated with KPL LumiGLO Chemiluminescent Substrate (SeraCare) and chemiluminescence was detected in ChemiDoc™ MP System (Bio-Rad). The detected bands were quantified in ImageLab 6.0.1 (Bio-Rad). To normalize the PSMA signal intensity between the 2 membranes, sample 22 was present on both membranes and used as an internal standard.

### 4.6. Immunohistochemistry

Immunohistochemical staining was performed on deparaffinized slides of 19 out of the 22 PPGL samples included in the study using standard manufacturer’s protocol on Ventana Benchmark Ultra automated immunostainer (Roche, Basel, Switzerland). Three samples (Nos. 4, 9, and 15) were not available for staining. The pretreatment was a 64-minute reagent retrieval with the CC1 (cell conditioning 1) reagent by Roche (catalog #950-224). The primary anti-PSMA antibody (clone 3E6) from DAKO/Agilent (catalog #M3620) was diluted 1:50 and incubated with the slides for 2 h. The signal was detected using an Ultraview Detection DAB Kit (Roche, catalog #760-500), which includes an HRP-conjugated secondary antibody and DAB substrate.

## 5. Conclusions

Integral membrane proteins represent roughly 25–30% of the human proteome. Their physical and chemical properties and low expression levels are to be blamed for their underrepresentation in standard trypsin-based proteomic analyses, requiring novel and combined strategies. Deep membrane proteome profiling of human PPGL and control adrenal medulla identified over 2100 IMPs. This number represents roughly one-third of all predicted human IMPs. To our knowledge, this is the most comprehensive dataset on the PPGL membrane proteome and also on solid tumor membrane proteomes in general. The unbiased analysis led to the first demonstration of PSMA overexpression in a subset of human PPGL. Identification and confirmation of PSMA overexpression in PPGL provides the proof of concept and, more importantly, opens the door to PSMA-based theranostics for Cluster 1 PPGLs.

## Figures and Tables

**Table 1:** Patient and sample information.

Patient No.	Sex	Tumor Type	PPGL Cluster	Mutated Gene	Used in Proteomics (P) and/or Western Blotting
1	F	PGL	1	*SDHB*	P,W
2	F	PGL	1	*SDHB*	P,W
3	M	PGL	1	*SDHB*	P,W
4	M	PGL	1	*SDHB*	W
5	M	PGL	1	*SDHB*	W
6	F	PGL	1	*SDHB*	W
7	M	PGL	1	*SDHB*	W
8	F	PGL	1	*SDHB*	W
9	F	PGL	1	*SDHB*	W
10	M	PHEO	1	*VHL*	W
11	F	PHEO	1	*VHL*	W
12	F	PHEO	1	*VHL*	W
13	M	PGL	1	*VHL*	P,W
14	M	PGL	1	*VHL*	P,W
15	F	PHEO	1	*EPAS1*	W
16	M	PHEO	2	*RET*	W
17	F	PHEO	2	*RET*	W
18	M	PHEO	2	*RET*	W
19	F	PHEO	2	*RET*	W
20	F	PHEO	NA	Sporadic	W
21	F	PHEO	NA	Sporadic	W
22	M	PHEO	NA	Sporadic	W

**Table 2:** Eighteen “tumor enriched” integral membrane proteins with cell surface localization were identified in all 5 PGLs but absent in the control adrenal medulla. “TM segments” indicates the number of predicted transmembrane segments/domains. Surface prediction consensus (SPC) scores of 3 or 4 indicate a high probability of cell surface localization.

Accession	Protein Name	Gene Name	TM Segments	SPC Score
A8MWY0	Endosome/lysosome-associated apoptosis and autophagy regulator family member 2	ELAPOR2	1	3
Q86T13	C-type lectin domain family 14 member A (Epidermal growth factor receptor 5)	CLEC14A	1	3
Q9Y5G0	Protocadherin gamma-B5	PCDHGB5	1	3
Q9UNN8	Endothelial protein C receptor (CD antigen CD201)	PROCR	1	4
Q04609	Glutamate carboxypeptidase 2, (Prostate-specific membrane antigen, PSMA)	FOLH1	1	3
Q96EP9	Sodium/bile acid cotransporter 4 (Solute carrier family 10 member 4)	SLC10A4	7	3
Q9ULK0	Glutamate receptor ionotropic, delta-1 (GluD1)	GRID1	3	3
Q13936	Voltage-dependent L-type calcium channel subunit alpha-1C	CACNA1C	24	3
O00391	Sulfhydryl oxidase 1	QSOX1	1	3
P80370	Protein delta homolog 1 (DLK-1)	DLK1	1	3
Q9NQX7	Integral membrane protein 2C (Cerebral protein 14)	ITM2C	1	3
P09619	Platelet-derived growth factor receptor beta (PDGF-R-beta) (CD antigen CD140b)	PDGFRB	1	4
Q9NPY3	Complement component C1q receptor (CD antigen CD93)	CD93	1	4
Q9H6X2	Anthrax toxin receptor 1 (Tumor endothelial marker 8)	ANTXR1	1	4
Q9H2H9	Sodium-coupled neutral amino acid transporter 1 (Solute carrier family 38 member 1)	SLC38A1	11	4
O75882	Attractin	ATRN	1	4
P17813	Endoglin (CD105)	ENG	1	4
Q9HCJ1	Progressive ankylosis protein homolog (ANK)	ANKH	8	3

## Data Availability

Proteomics data are freely available via ProteomeXchange with identifier PXD029344.

## Supplementary Materials

The following are available online. Figure S1: The overlaps in numbers of identified proteins among the four Pitchfork methods, and the distribution of identified IMPs according to the numbers of their transmembrane segments; Figure S2: Annotated MS/MS spectra of PSMA; Table S1: Detailed patient and sample information; Table S2: The full list of identified integral membrane proteins; Table S3: List of 45 “tumor enriched” IMPs present in all 5 PPGLs but not detected in the control tissue.

## Sample Availability

Samples of the compounds are not available from the authors.
