# Supplementary material for: Deep Membrane Proteome Profiling Reveals Overexpression of Prostate-Specific Membrane Antigen (PSMA) in High-Risk Human Paraganglioma and Pheochromocytoma, Suggesting New Theranostic Opportunity
Source: Molecules. 2021 Oct 29;26(21):6567. doi: 10.3390/molecules26216567 (PMC8587166; doi:10.3390/molecules26216567)
Supplement: Supplementary file 1 [file molecules-26-06567-s001.zip › supplementary figure 1.pdf]

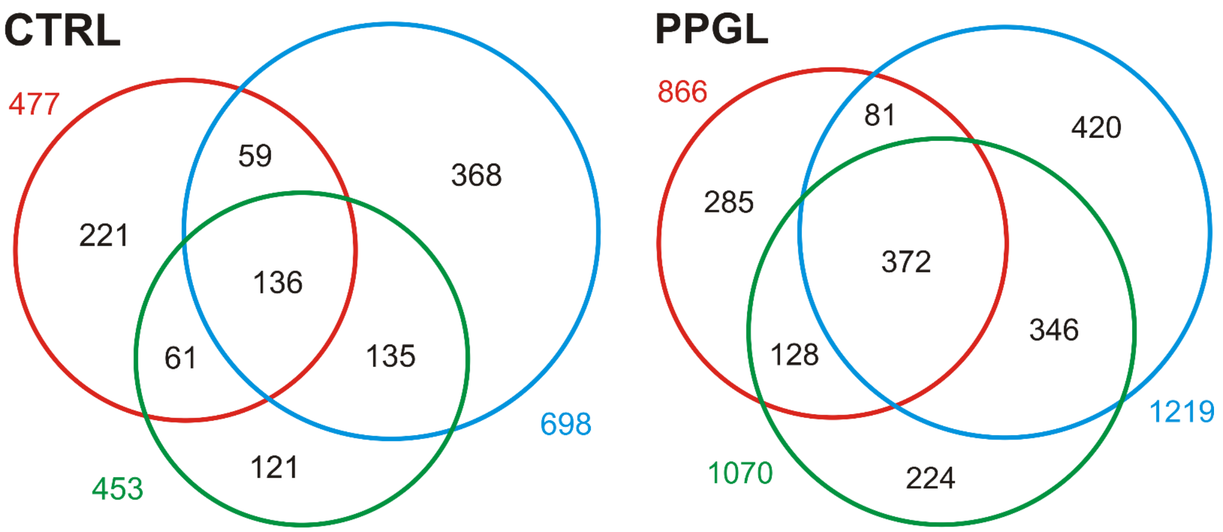

Venn diagrams of IMPs identified in CTRL and using hpTC (red), SDC-trypsin (green) and N-glycocapture methods, i.e. N-glyco FASP and SPEG (blue, taken together for simplicity of Venn diagrams)

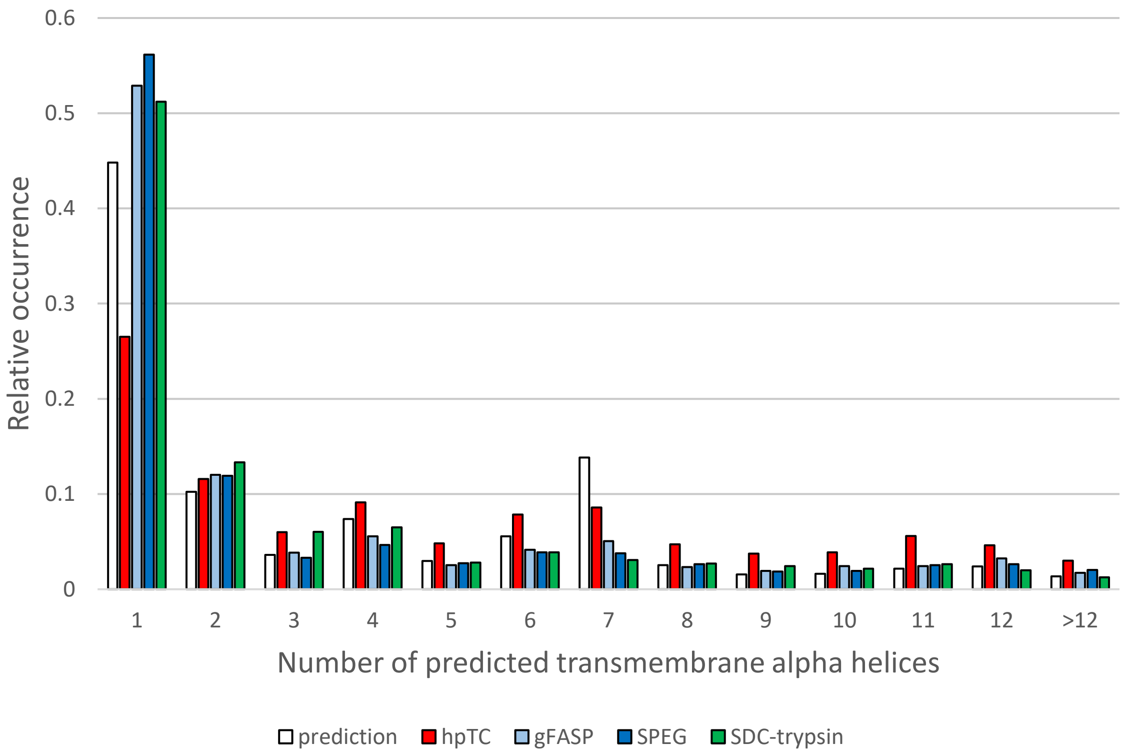

Predicted transmembrane alpha-helical segments in the identified IMPs using the four Pitchfork methods. Prediction of whole-proteome distribution of the number of predicted transmembrane segments is added for comparison. The prediction of transmembrane domains in the identified proteins and in the whole human proteome was done using the Tied Mixture Hidden Markov Model (TMHMM) method
