# Supplementary material for: Deep Membrane Proteome Profiling Reveals Overexpression of Prostate-Specific Membrane Antigen (PSMA) in High-Risk Human Paraganglioma and Pheochromocytoma, Suggesting New Theranostic Opportunity
Source: Molecules. 2021 Oct 29;26(21):6567. doi: 10.3390/molecules26216567 (PMC8587166; doi:10.3390/molecules26216567)

Raw File	Scan	Method	Score	m/z
Patient14_trypsin	89072	ITMS; CID	73.72	780.73

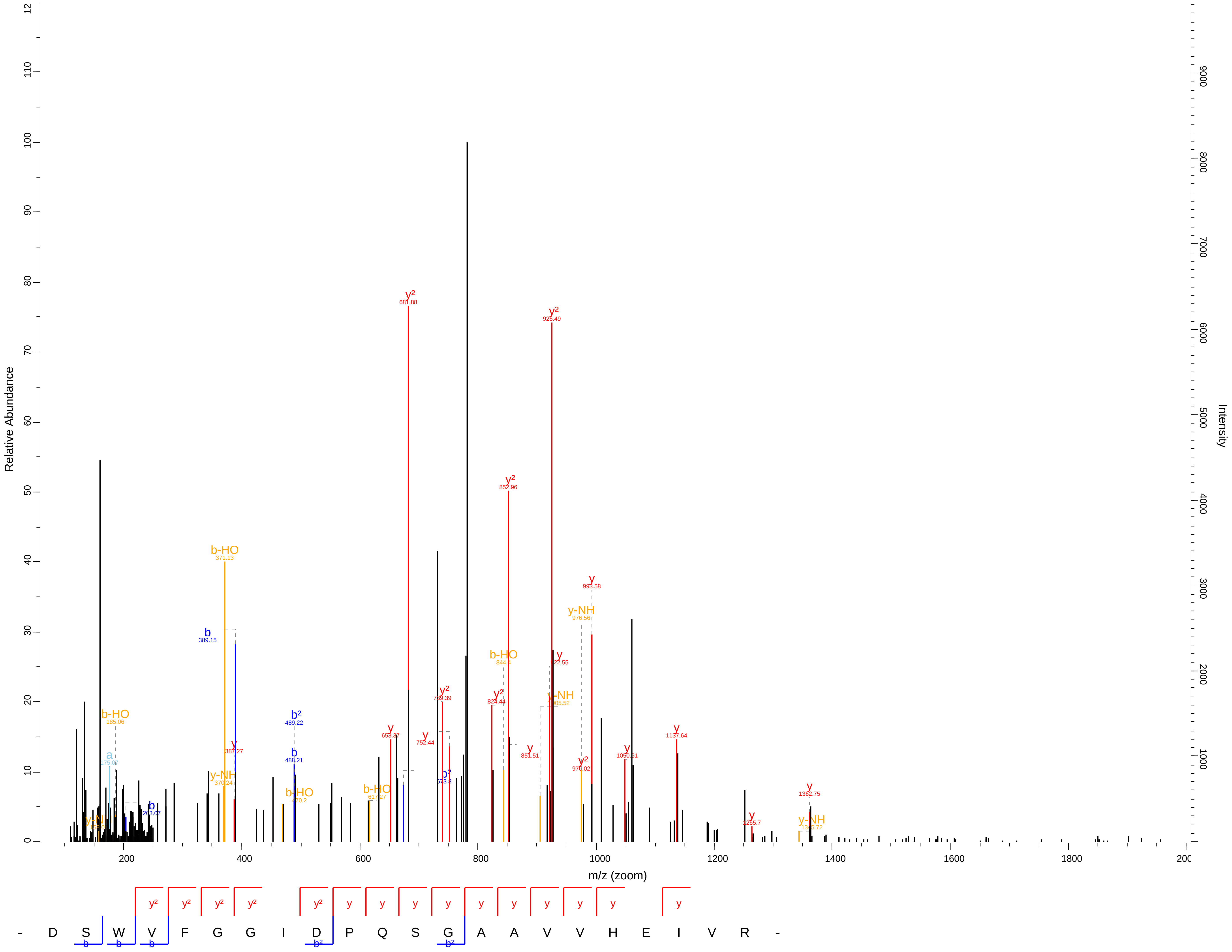

Raw File	Scan	Method	Score	m/z
Patient1_gfasp	67771	ITMS; CID	55.57	861.74

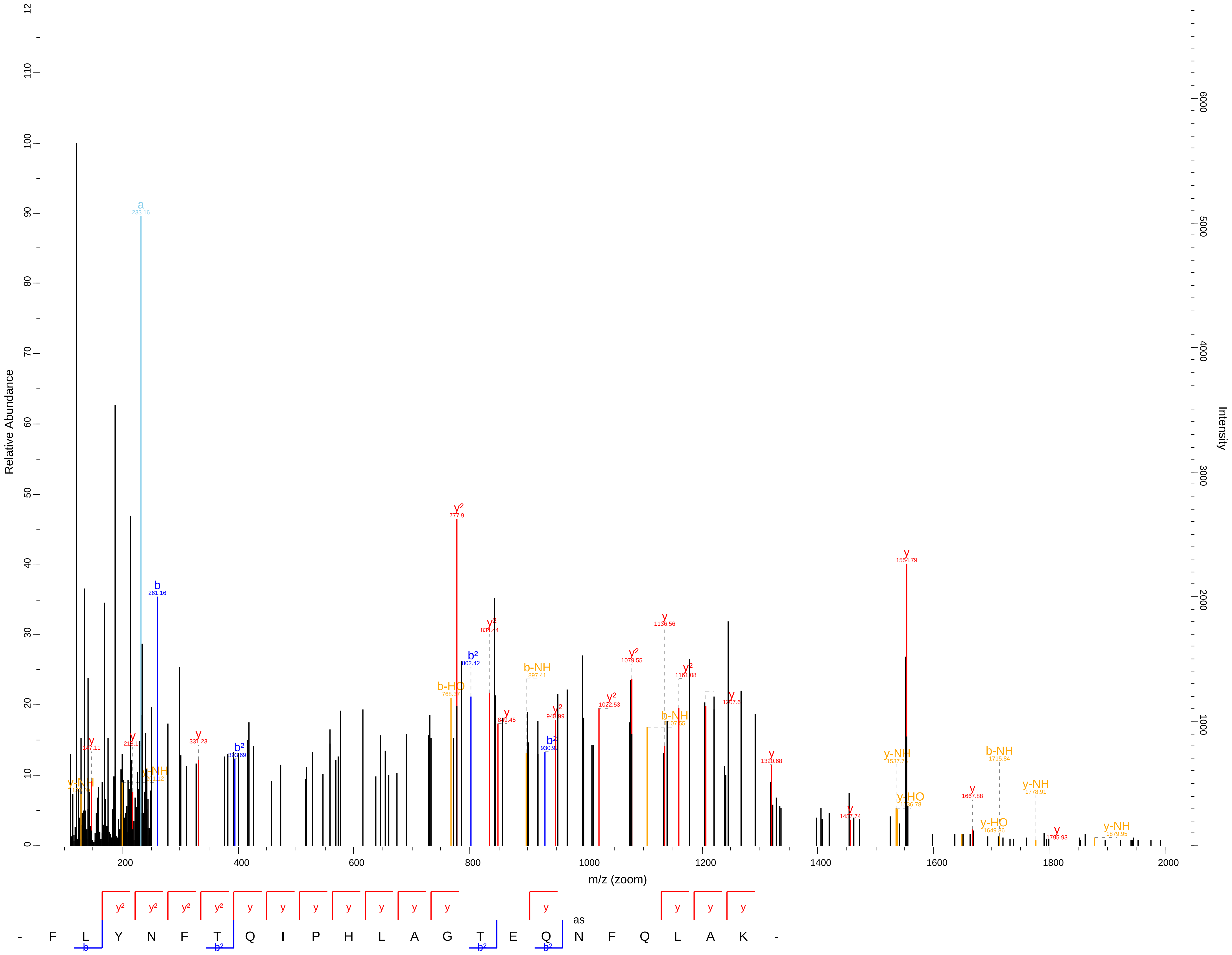

Raw File	Scan	Method	Score	m/z
Patient14_gfasp	75164	ITMS; CID	79.31	861.11

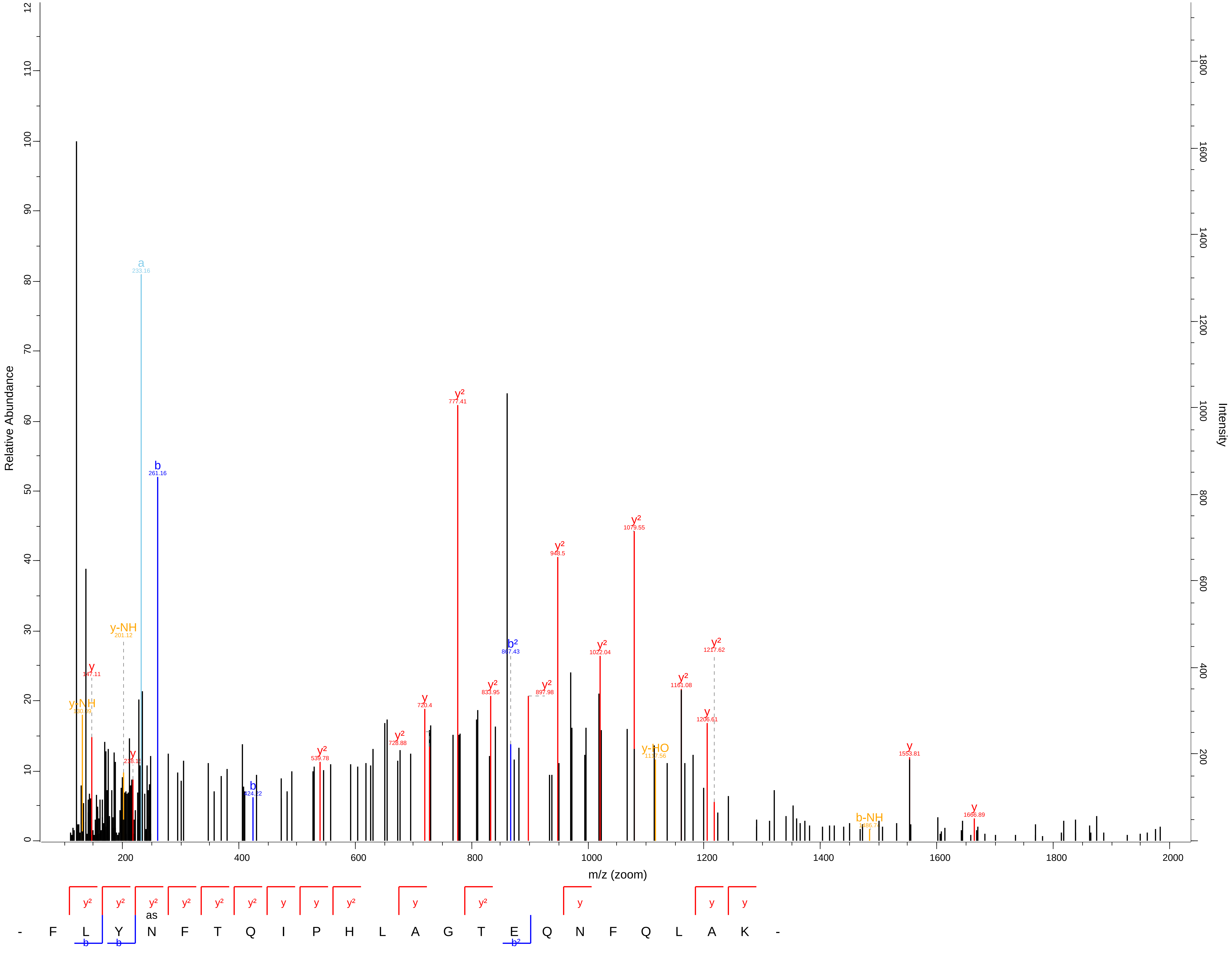

Raw File	Scan	Method	Score	m/z
Patient3_speg	8484	ITMS; CID	48.8	1264.05

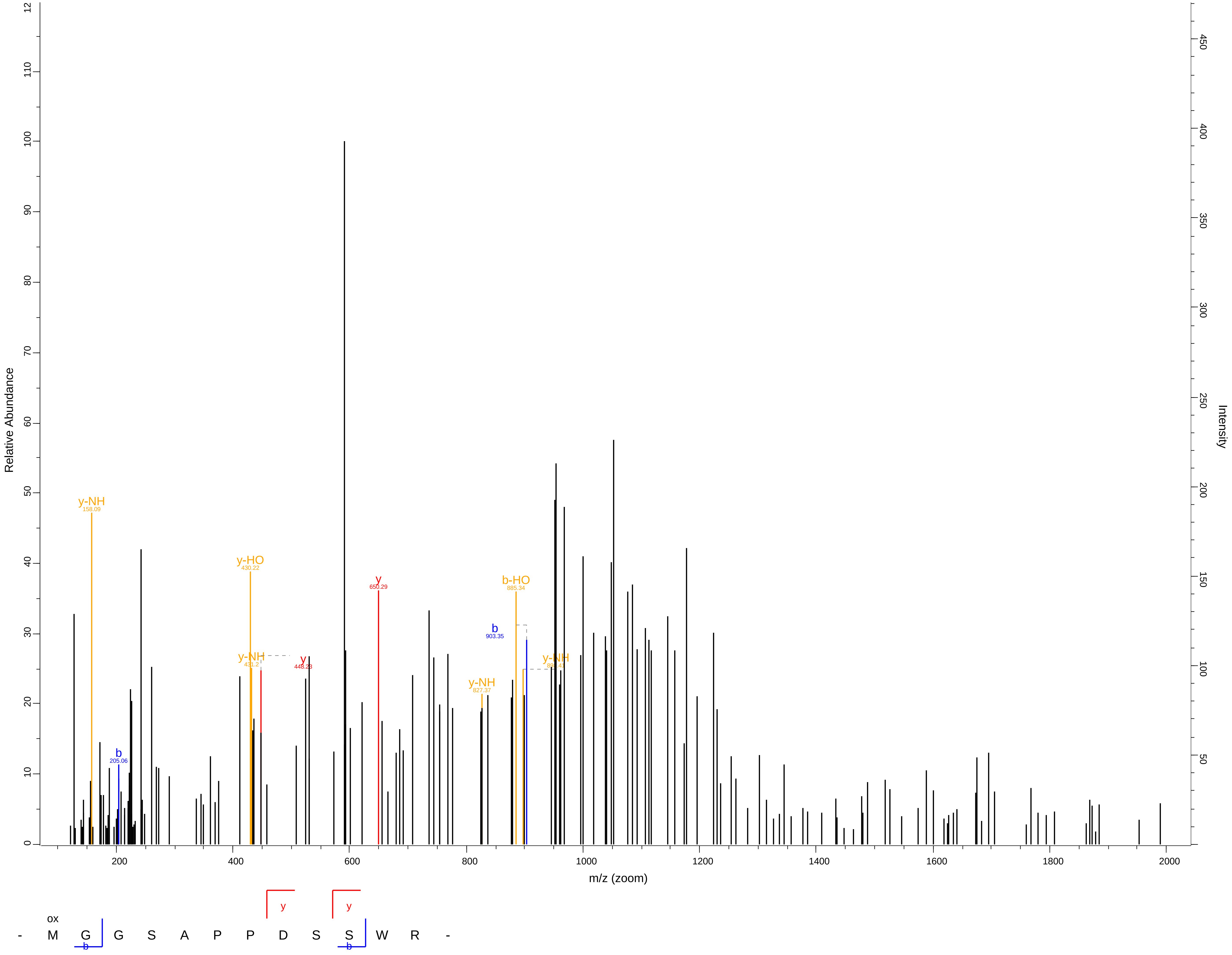

Raw File	Scan	Method	Score	m/z
Patient1_gfasp	65599	ITMS; CID	96.1	631.31

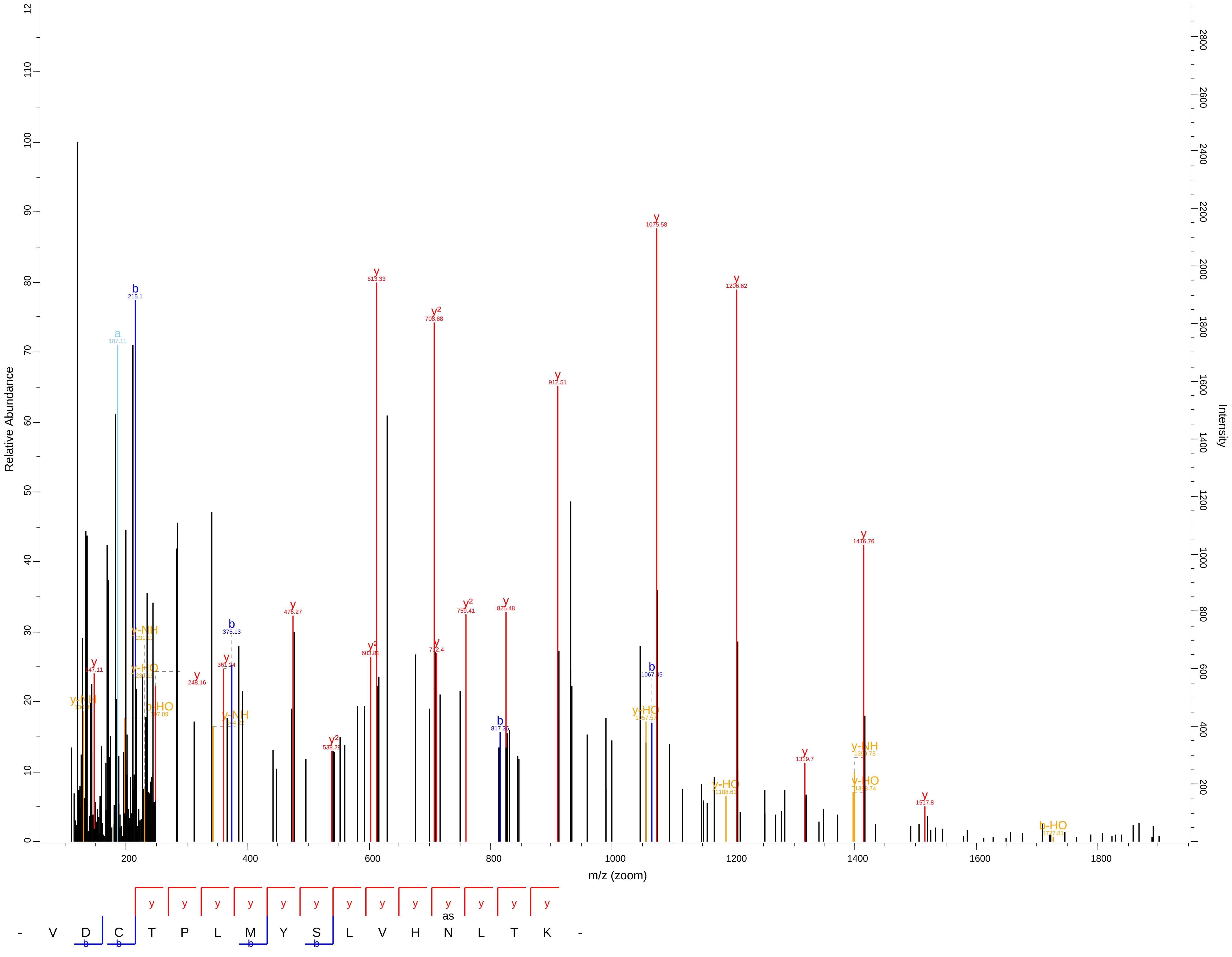

Raw File	Scan	Method	Score	m/z
Patient2_gfasp	69688	ITMS; CID	79.84	631.31

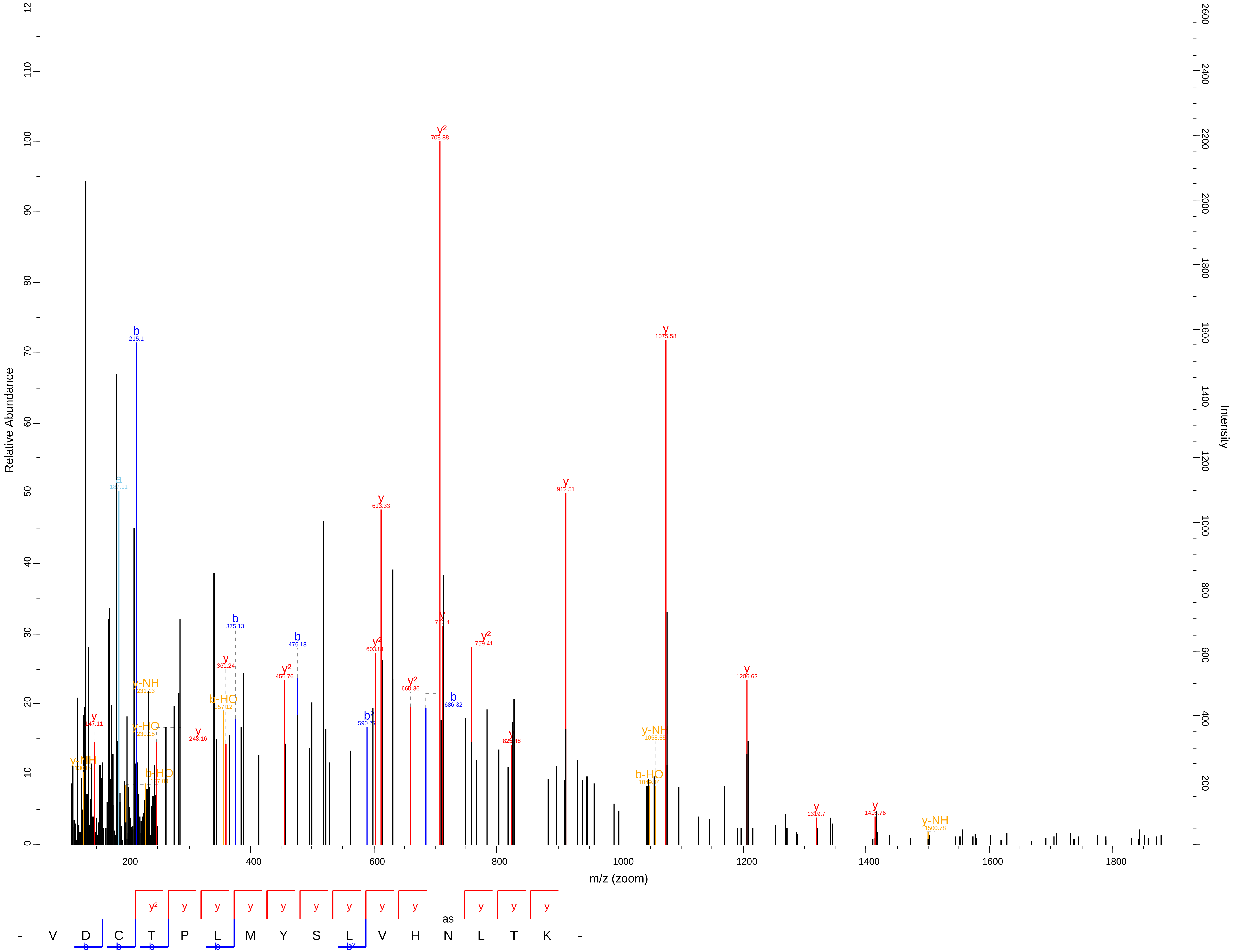

Raw File	Scan	Method	Score	m/z
Patient3_speg	63282	ITMS; CID	74.34	636.98

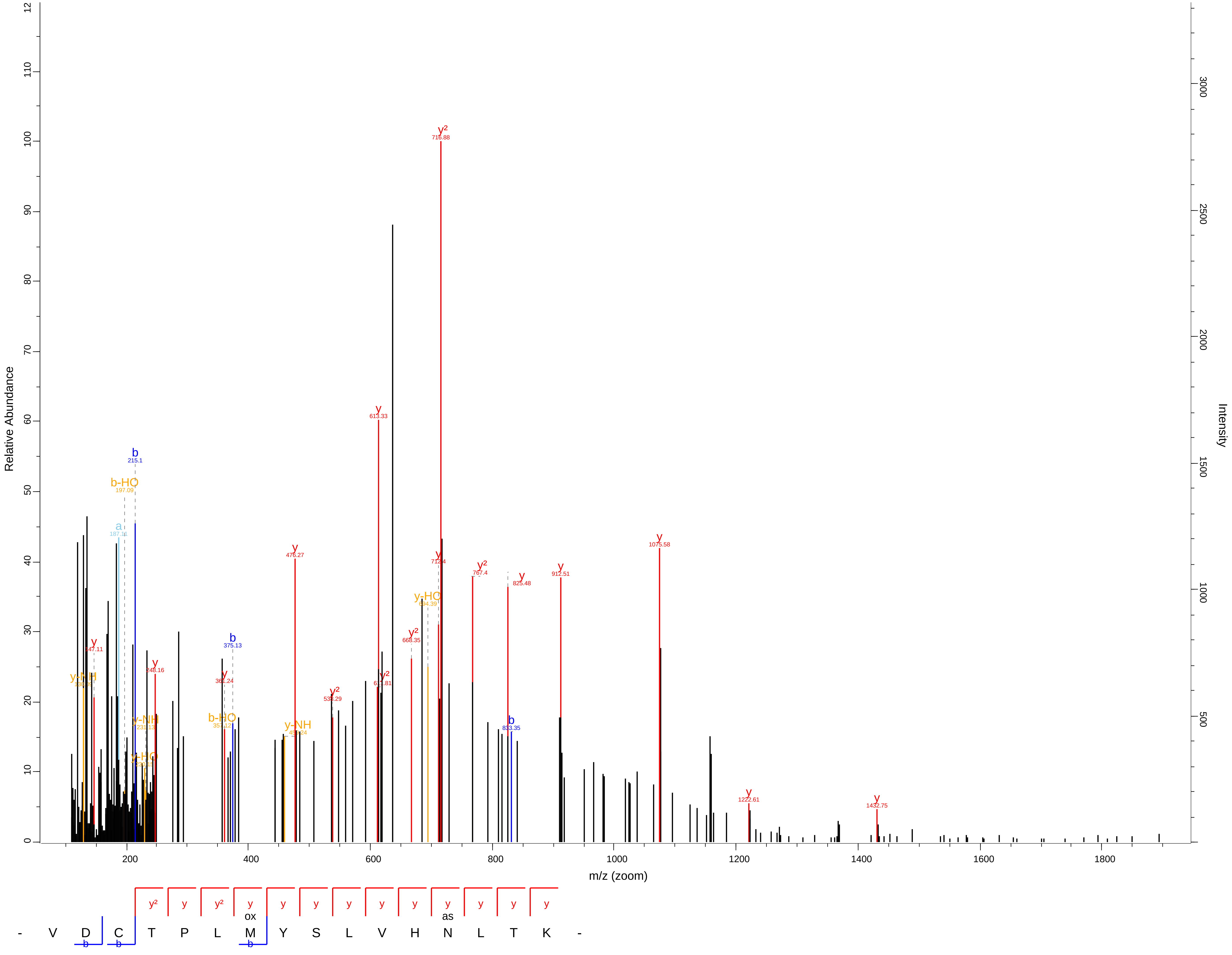

Raw File	Scan	Method	Score	m/z
Patient3_speg	58630	ITMS; CID	85.27	907.44

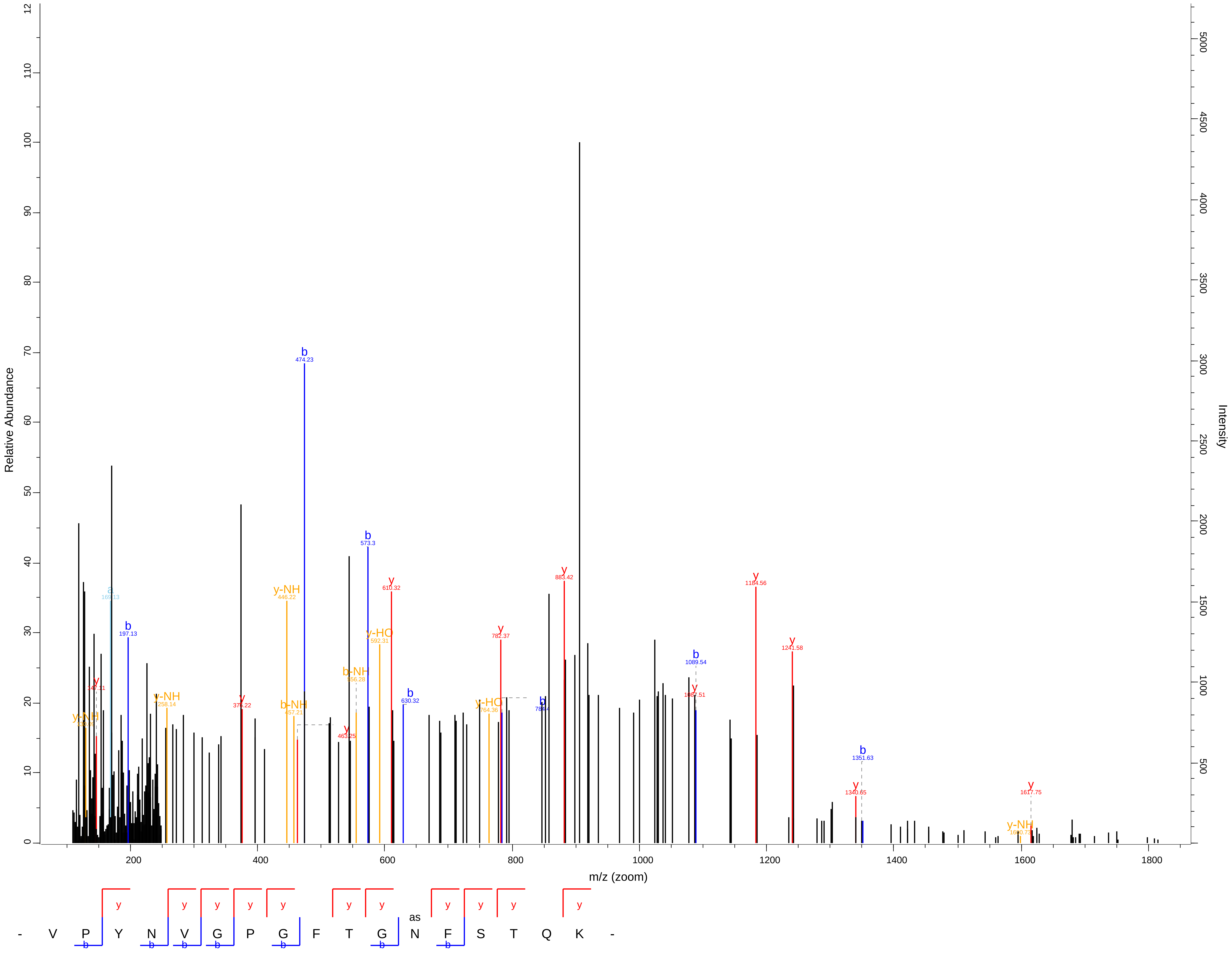

Raw File	Scan	Method	Score	m/z
Patient14_gfasp	47419	ITMS; CID	153.49	907.44

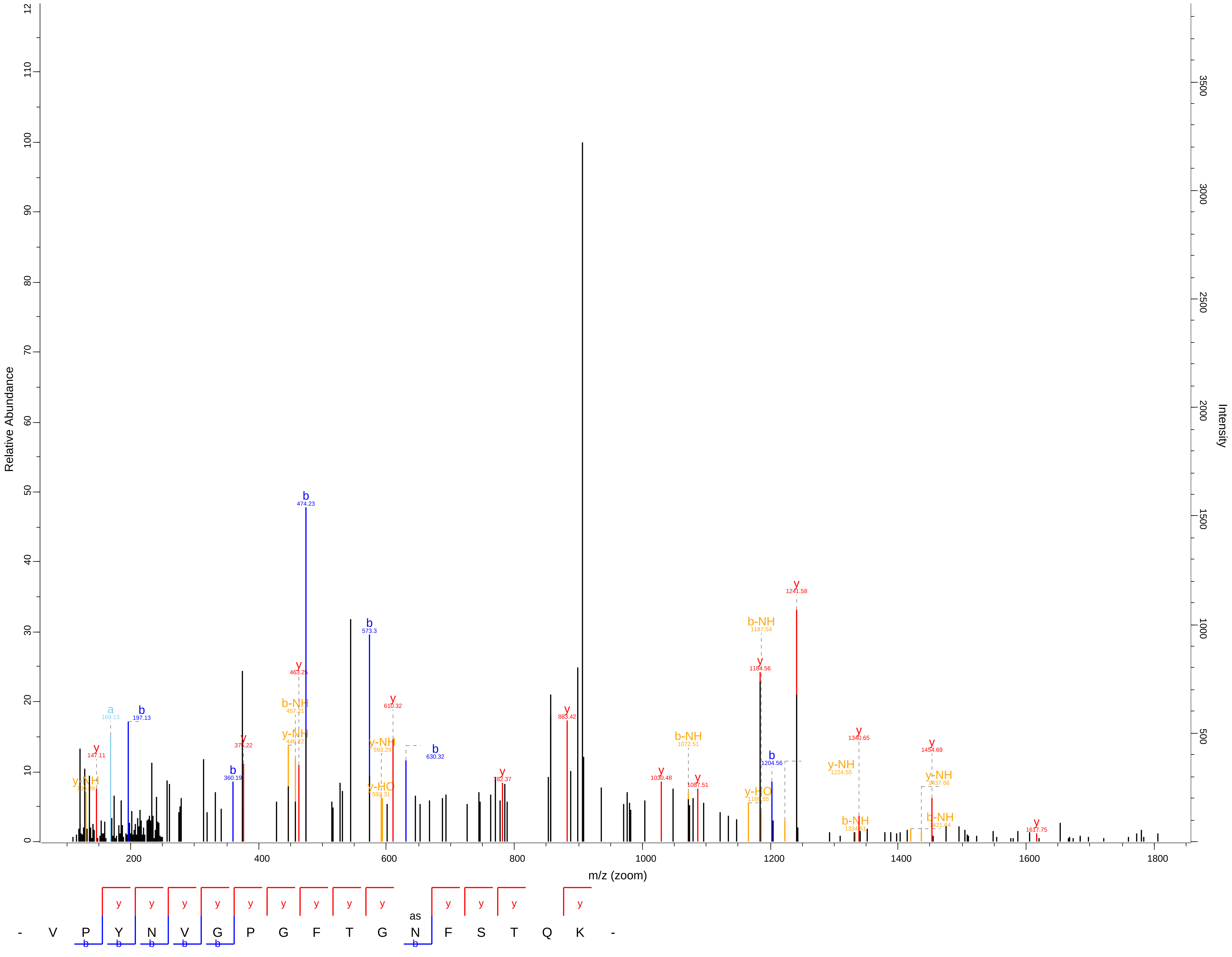

Raw File	Scan	Method	Score	m/z
Patient14_gfasp	47558	ITMS; CID	99.84	907.94

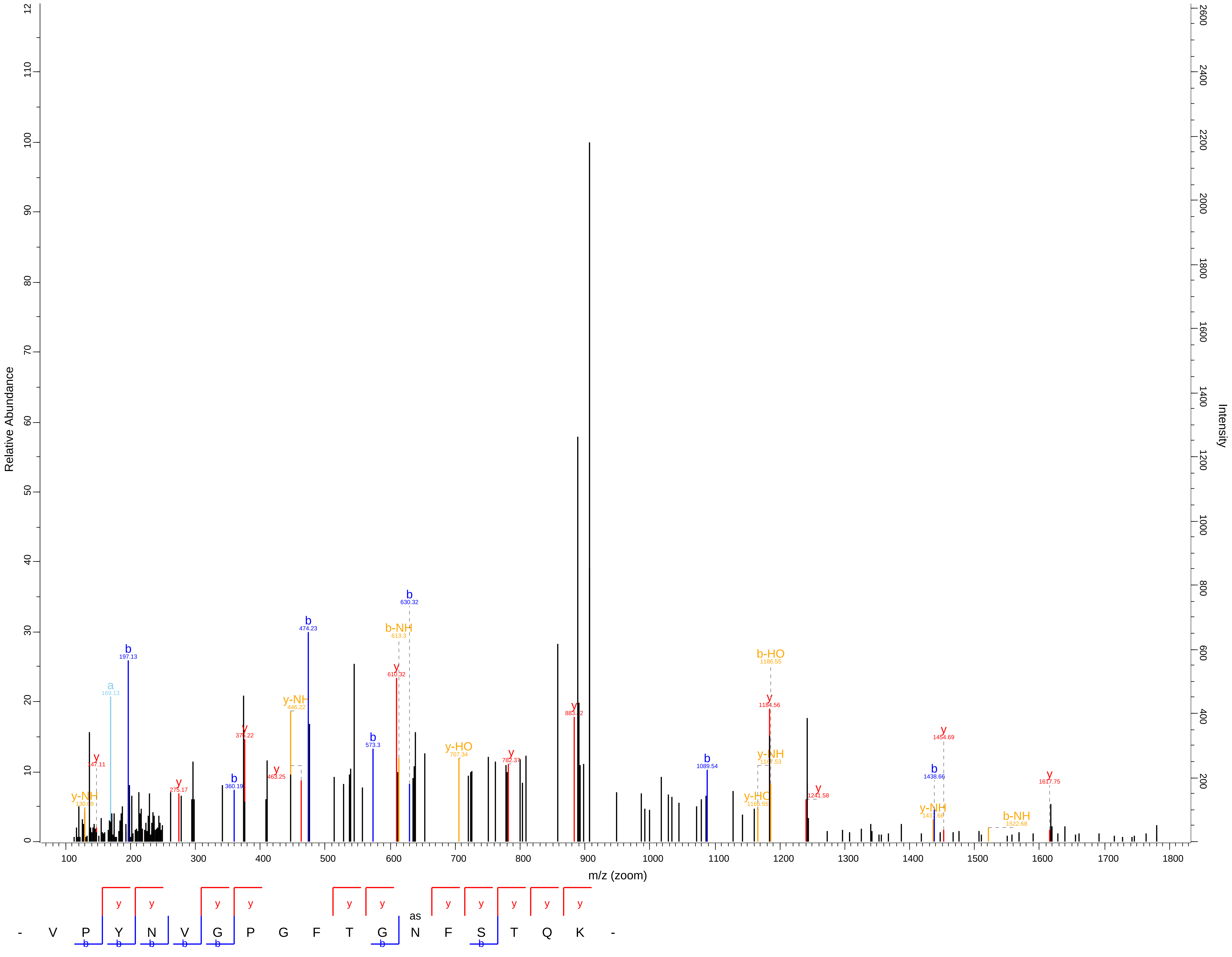

Raw File	Scan	Method	Score	m/z
Patient13_gfasp	42208	ITMS; CID	79.47	907.94

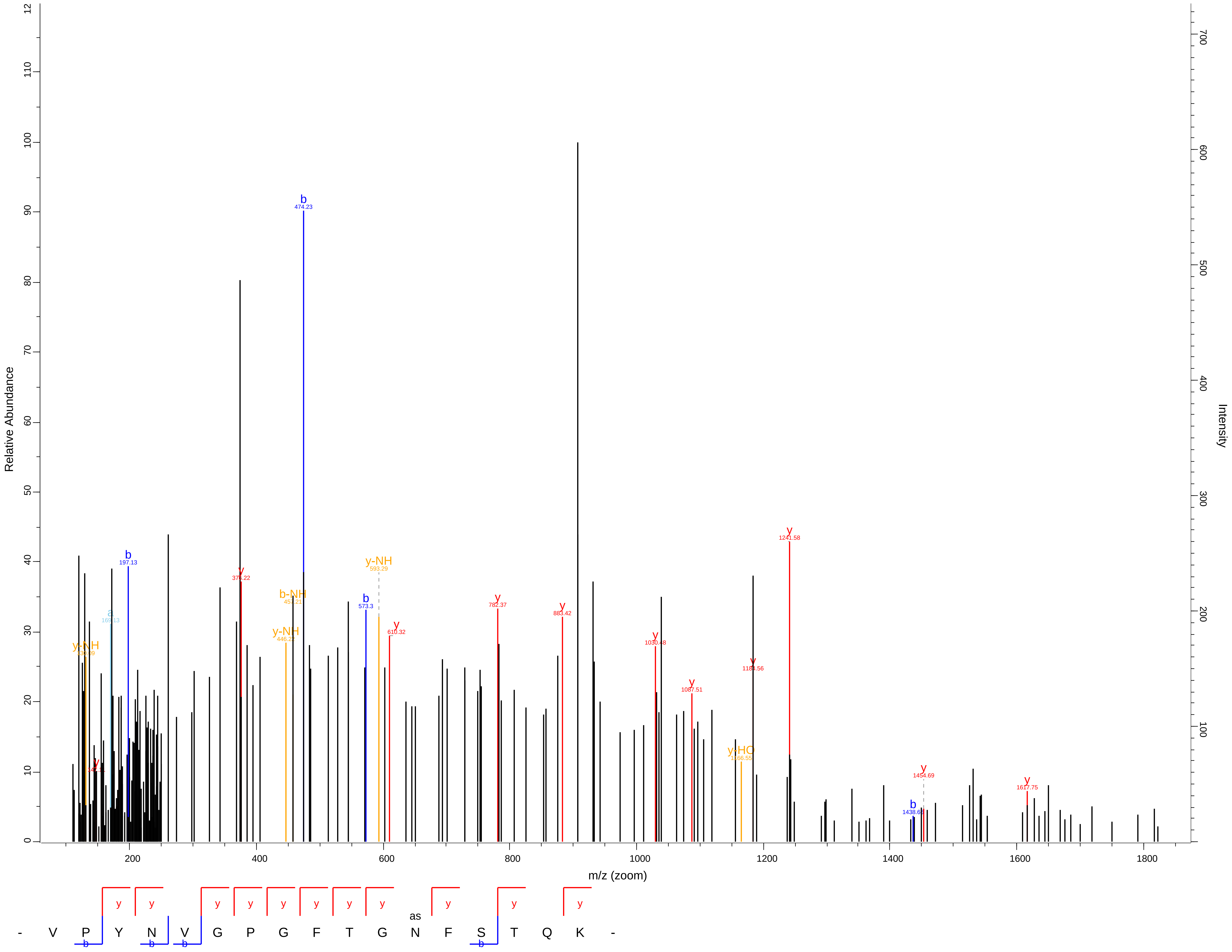

Supplement: Supplementary file 1 [file molecules-26-06567-s001.zip › supplementary figure 2.pdf]
